# If I tweet will you cite later? Follow-up on the effect of social media exposure on article downloads and citations

**DOI:** 10.1007/s00038-020-01519-8

**Published:** 2020-11-07

**Authors:** Thomy Tonia, Herman Van Oyen, Anke Berger, Christian Schindler, Nino Künzli

**Affiliations:** 1 Institute of Social and Preventive Medicine, University Bern, Bern, Switzerland; 2 Epidemiology and Public Health, Sciensano, Brussels, Belgium; 3 Public Health and Primary Care, UGent, Gent, Belgium; 4 Swiss Tropical and Public Health Institute, Basel, Switzerland; 5 University of Basel, Basel, Switzerland

**Keywords:** Social media, Citations, Downloads, Bibliometrics, Twitter, Facebook

## Abstract

**Objectives:**

We previously reported that random assignment of scientific articles to a social media exposure intervention did not have an effect on article downloads and citations. In this paper, we investigate whether longer observation time after exposure to a social media intervention has altered the previously reported results.

**Methods:**

For articles published in the International Journal of Public Health between December 2012 and December 2014, we updated article download and citation data for a minimum of 24-month follow-up. We re-analysed the effect of social media exposure on article downloads and citations.

**Results:**

There was no difference between intervention and control group in terms of downloads (*p* = 0.72) and citations (*p*= 0.30) for all papers and when we stratified by open access status.

**Conclusions:**

Longer observation time did not increase the relative differences in the numbers of downloads and citations between papers in the social media intervention group and papers in the control group. Traditional impact metrics based on citations, such as impact factor, may not capture the added value of social media for scientific publications.

**Electronic supplementary material:**

The online version of this article (10.1007/s00038-020-01519-8) contains supplementary material, which is available to authorized users.

## Introduction

In a previously published randomised controlled study (Tonia et al. 2016), we investigated the effect of an experimental social media (SM) exposure of scientific papers on subsequent article downloads and citations. For the previous analysis, we assessed the number of article downloads and citations over a period that ranged between 3 and 27 months (mean 407.67 days; range 90–821 days) and found no effect of the experimental SM exposure on the number of downloads (*p* = 0.60) and citations (*p* = 0.88). However, the observation period for some papers was too short, limiting the statistical power of the study and possibly biasing some estimates. Observation time is especially relevant for citations: citations cumulate over time and IJPH papers, in particular, are cited more in the second year after their publication compared to the first. It has also been indicated that the lifetime value of blog posts might be longer than commonly thought and reach up to 700 days (IZEA 2015). Moreover, writing a paper takes time and authors might save a paper they have seen on social media with the intention of citing it later on. As a result, we decided to update our analyses and present the results for a later time point, when all the included papers would have a follow-up period of at least 24 months. We were interested to see whether an intervention effect, which could not be seen in the previous analysis, might have emerged with longer observation time.

## Methods

Detailed methods of the trial have been previously reported (Tonia et al. 2016). In brief, all original articles published in the International Journal of Public Health between December 2012 and December 2014 were randomised to a standardised SM exposure (blog post, Twitter and Facebook). The Twitter and Facebook posts contained a title or summary of the study with a link and relevant hashtags when applicable; whenever possible, authors of papers that had Twitter accounts were tagged on the Twitter post. The blog post was longer than Twitter and Facebook posts and contained more details on the paper. Any reaction received as a result of these posts was replied upon. The SM exposure was applied at three different time points after online publication (immediately after; 2 weeks after first exposure; and 10 weeks after second exposure) or no exposure (control group). We then analysed the effect of the SM exposure intervention on article downloads and citations, starting from randomisation and until December 2014, also adjusting for length of observation time and papers’ geographical origin. We also presented the results stratified by whether the paper was published open access or not.

We repeated our analyses with updated data on article downloads and citations up to the end of December 2016, thus extending the observation period by two years. The two endpoints remained the same, namely (1) the number of full-text article downloads, as provided by our publisher, Springer, and (2) the number of article citations, by using data from Web of Science Core Collection (Clarivate Analytics). We defined the online publication date of the citing article as the date of citation. We considered citations until 31 December 2016. We also evaluated the possible effect of open access status on both downloads and citations.

### Statistical analysis

We used the Wilcoxon rank sum test to determine differences in quantitative variables between the two groups. Correlations between quantitative variables were assessed using Spearman’s rank correlation coefficient; Rate ratios of downloads and citations between intervention and control group were estimated using negative binomial regression models for download and citation counts with group as independent variable. Length of observation period of the paper was used as offset. To display the time course of downloads and citations as a function of time since publication, download and citation data were aggregated into 4-week intervals after publication. For more details into the remaining analyses, please see the previous publication (Tonia et al. 2016).

## Results

A total of 130 papers were analysed (*n* = 65 each in the intervention and the control groups). Details on study sample characteristics can be found in Table 1 of the previous publication (Tonia et al. 2016). At the time of the present analysis, the mean follow-up time of the papers since exposure to the intervention was 1049 days (range 731–1462 days).

**Table 1:** Estimated incidence rate ratios (IRR) of citations and downloads associated with randomised controlled social media exposure of original articles published between December 2012 and December 2014 in the International Journal of Public Health

	IRR^a^	95%-CI		*p* value
*Number of downloads*				
Unadjusted	1.04	0.84	1.28	0.72
After adjustment for region^b^	1.07	0.86	1.32	0.56
Region 1	1.06	0.80	1.40	
Region 2	1.02	0.63	1.64	0.96*
Region 3	1.12	0.70	1.81	
W/o open access	1.12	0.83	1.21	1
*Number of citations*				
Unadjusted	1.16	0.88	1.54	0.30
After adjustment for region^b^	1.16	0.87	1.56	0.32
Region 1	1.35	0.93	1.94	
Region 2	0.74	0.39	1.39	0.28*
Region 3	1.17	0.59	2.32	
W/o open access	1.09	0.82	1.44	0.56

*p value of interaction between region and social media coverage

^a^Incidence rate ratio

^b^Region 1 = Europe, region 2 = North America, Australia, New Zealand, region 3 = Africa, Asia, South America

### Effects of SM exposure on downloads

**Fig. 1:**
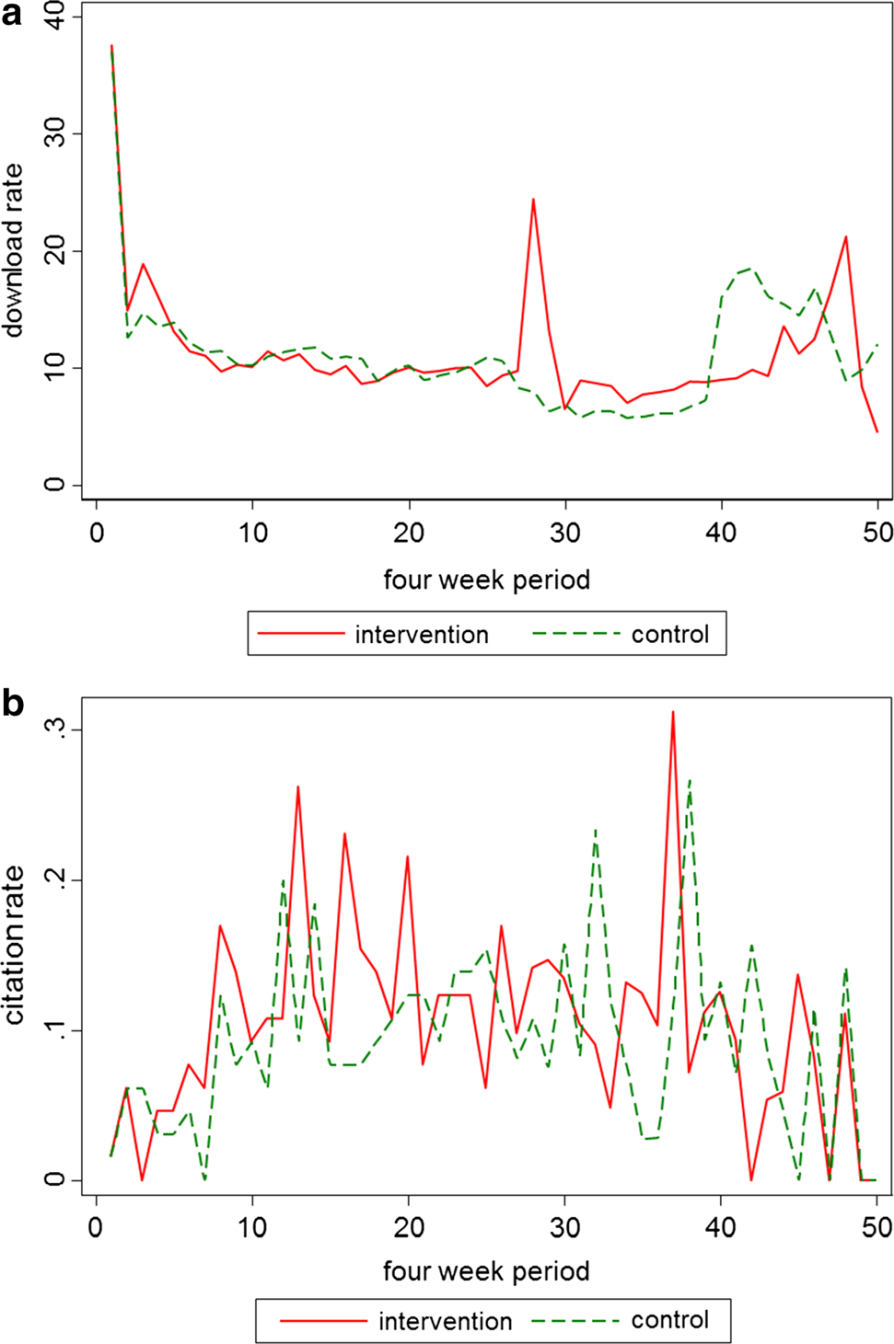
Download (**a**) and citation rate (**b**) by time (4 week periods) since publication of original articles published between December 2012 and December 2014 in the International Journal of Public Health; rate was defined as an average number of citations / downloads per paper under observation in the respective four week period

There were 55,308 downloads: 27,812 in the SM exposure group and 27,496 in the control group. The mean number of downloads per paper was 427.9 (SD 345.5, median 312, range 153–1932) for the SM exposure group and 423.0 (SD 324.1, median 314, range 136–1655) for the control group. The number of downloads did not differ significantly between groups (*p* = 0.84, Wilcoxon rank sum test). Figure 1a shows the evolution of number of downloads over time, in 4-week intervals after publication for the two groups. Overall, the time course of downloads was quite similar in the two groups. There was, however, a peak in downloads for the intervention group around 110 weeks after publication. There was one paper that was responsible for this peak (Nuutinen et al. 2014); this paper had become available to download for free for 4 weeks during this period, which might explain this increase in the number of downloads. Figure 1 also shows a peak in downloads for both groups between months 40 and 50. Due to the smaller number of papers under observation after 40 weeks, the uncertainty of the estimates in this period is big, so we cannot draw any conclusions for this peak.

Table 1 shows the results of negative binomial regression analyses. The rate ratio (RR) of downloads between SM exposure and control group was 1.04 (95% CI 0.84–1.28; *p* = 0.72; incidence = 0.416/day vs. 0.396/day). Adjusting for the corresponding author’s region of origin did not alter the rate ratio (RR) 1.07 (95% CI 0.86–1.32; *p* = 0.56). When we ran the model with separate intervention effect variables for the three main regions, it returned very similar intervention effect estimates for the three regions.

### Effects of SM exposure on citations

During the follow-up period for the 130 manuscripts, there were 504 citations: 267 in the SM exposure group and 237 in the control group. The mean number of citations per paper was 4.11 (SD 3.88; median 3; range 0–21) for the SM exposure group and 3.65 (SD 2.93; median 3; range 0–12) for the control group. The difference in the number of citations was not statistically significant between the two groups (*p* = 0.70, Wilcoxon rank sum test). Figure 1b shows that the time evolution of the number of citations in 4-week intervals was similar in the two groups.

Table 1 shows the results of negative binomial regression analyses. The rate ratio of citations between exposure and control group was 1.16 (95% CI 0.88–1.54, *p* = 0.30; incidence = 0.0040/day vs. 0.0034/day). Adjusting for region of origin did not change the rate ratio (RR 1.16, 95% CI 0.87–1.56, *p* = 0.32). Running the model with separate intervention effect variables for the three main regions of origin of the corresponding author, we found that the intervention was positively associated with the incidence rate of citations in regions 1 and 3, while the incidence rate ratio was 0.74 in region 2. This association, however, was not statistically significant (*p* = 0.28).

### Influence of open access status

We found 10,392 downloads for the nine open access articles (mean download per article = 1154.7, median = 1223, SD 515.6) and 44,916 for the 121 non-open access articles (mean = 371.2 median = 294, SD 243.2; *p* < 0.0001, Wilcoxon rank sum test).

The distribution of the number of citations did not significantly differ between open access papers (mean: 6, median: 5, SD 6.02, total number of citations: 54) and non-open access papers (mean: 3.72, median: 3, SD 3.14, total number of citations: 450; *p* = 0.17, Wilcoxon rank sum test). When we stratified analyses by open access status, rate ratios of downloads between intervention and control group were similar for open and non-open access journals, while the rate ratio of citations was higher among open access journals (2.69, 95% CI 0.99–7.28, n = 9, *p* = 0.051) than among non-open access journals (1.09, 95% CI 0.82–1.45, *n* = 121, *p* = 0.56) (Online Supplement).

### Correlations

Later publication date shortened the length of time between publication and the end of our study. As expected, this was associated with fewer downloads and citations. The number of downloads and the number of citations significantly correlated for all papers (Spearman’s rho = 0.32, *p* = 0.0002). It was stronger in the SM exposure group (rho = 0.47, *p* = 0.0001) than in the non-exposure group (rho = 0.19, *p* = 0.13; Online supplement), this difference was not statistically significant (*p* = 0.07, permutation test).

## Discussion

There were no relative differences in the numbers of downloads and citations between papers having been observed for at least two years after being exposed to SM and those without SM exposure having been observed for the same time. The results from the updated analyses with extended observation period after exposure were very similar to the ones of the original analyses. If anything, the CIs became narrower in general. We can, therefore, conclude that increased time of observation after the SM intervention did not increase the relative differences in numbers of downloads or citations between the two groups, even if there has been enough time for all the papers to have been cited.

Since our previous article, several studies have been published that looked at different aspects of the use of SM in scientific publishing. Most of them were observational studies reporting correlations. Some found positive correlations between online mentions and citations (Evaniew et al. 2017; Finch et al. 2017; Knight 2014; Peoples et al. 2016; Quintana and Doan 2016; Xia et al. 2016), while others found only small or no correlations (Cardona-Grau et al. 2016; Delli et al. 2017; Hébert et al. 2017; Livas and Delli 2017; Patthi et al. 2017; Peters et al. 2016; Rosenkrantz et al. 2017) or mixed results (Dal-Ré and Mahillo-Fernández, 2018). Similarly, some studies found positive correlations between number of followers, journal impact factor and number of citations (Cosco 2015), presence of Twitter and rise in impact factor (O'Kelly et al. 2017) or Altmetric score and impact factor (Scotti et al. 2017). The Altmetric score, however, would include any SM activities by the journal, so this result is difficult to interpret. In addition, it is not SM specific as it is a weighted score of different sources of attention a manuscript received. Looking from a journal perspective, some studies found that journals with a SM account scored higher on academic metrics (Alotaibi et al. 2016) or had significantly higher Altmetric scores for their articles (Wang et al. 2017); This latter study, however, found no overlap between trending articles and most cited ones. It is worth mentioning that even studies reporting positive correlations between SM use and citations draw attention to the fact that the correlations were rather modest (Xia et al. 2016) or that SM activity was not as strong a predictor of citation rates as other factors (Peoples et al. 2016). A meta-analysis (Bornmann 2015) of the correlation coefficients reported by studies having measured the correlation between Twitter citations and traditional citations found strong heterogeneity across studies; higher coefficients were reported mostly by studies with low case numbers and the meta-estimate obtained was close to 0, indicating that there is no relevant correlation between Twitter counts and traditional citation counts. The same study found a low correlation (*r* = 0.12) between blog counts and traditional citations and a medium to large correlation between bookmark counts from online reference managers and citation counts (*r* = 0.23 for CiteULike and *r* = 0.51 for Mendeley). Online reference managers have been found to have stronger correlations with citations in other studies as well (Rosenkrantz et al. 2017; Ruano et al. 2018).

Since our previous publication, we could not identify another RCT that looked at the effect of SM on citations. There were, however, two RCTs that looked at SM exposure and its effect on article page views (Adams et al. 2016; Fox et al. 2016b). A previously randomised controlled study by the same authors of the Fox study (Fox et al. 2014) did not find any effect of social media exposure on article views. In the new trial (Fox et al. 2016b), the authors tested the effects of an intervention of increased intensity of SM exposure and found no difference in 30-day (*p* = 0.38) and 7-day (*p* = 0.17) page views. The increased intensity of the intervention as well as the bigger number of Twitter and Facebook followers did not seem to change the results from the previous trial. In the second study (Adams et al. 2016), reviews from the Schizophrenia Cochrane group were randomised to SM exposure (Twitter and Weibo) versus no exposure. The study reports that reviews in the intervention group had more web page visits in one week compared to control (IRR 2.7; 95% CI 2.2–3.3); in addition, users spent more time viewing the intervention reviews.

We and other researchers have previously suggested that the number of citations and social media attention scores are measures of different types of impact (Haustein et al. 2014; Xia et al. 2016). Although some authors have suggested that higher quality research receives more mainstream attention (Cosco 2015), other authors have also indicated that high Altmetric scores might be influenced by an article’s novelty and public engagement and not necessarily by its impact on the scientific field (Wang et al. 2017). Another study found that opinion articles received relatively high SM interest in relation to their citation counts (Dal-Ré et al. 2017). Moreover, qualitative analysis suggests that article topics discussed in SM are more likely to relate to the more controversial and emotive areas (Knight 2014). As a result, predicting scientific success based on SM activity may not be appropriate (Ruano et al. 2018). Simple counting of online mentions without taking into account the content can lead to wrong conclusions, for instance in case of scientific misconduct that might receive a lot of SM attention (Bornmann and Haunschild 2018) or in case of automated software used for SM or single use tweets and duplicate tweets (Robinson-Garcia et al. 2017). Moreover, simply posting a title and a link to a publication is probably not as efficient as using the full possibilities of social media, such as hashtags, mentions and discussion threads. Future studies should focus on the different possibilities social media offer and how these and other characteristics such as the lifetime value of social media posts affect the outreach of papers.

To date, two years after its publication and after having received substantial SM exposure, our paper reporting the original results of our RCT (Tonia et al. 2016) has an Altmetric score of 226, reaching more than 1,000,000 (upper bound) Twitter followers. Nevertheless, the number of downloads (*n* = 1100) and citations (*n* = 6), albeit above the median, is still within the range for the papers that were included in the intervention group (median for downloads: 312; range 153–1932; median for citations: 3; range 1–9). The fact that the number of Twitter and Facebook followers of our Journal’s social media account has substantially increased since the original analysis (Facebook: from 399 to 2960 followers; Twitter from 1845 to 4236 followers) does not seem to have played a favourable role.

As a conclusion, we agree with other authors that SM presence can be beneficial for scientific papers and journals, but there is no evidence that SM presence will increase citations (Fox et al. 2016a; Peoples et al. 2016). Traditional impact measures are being increasingly challenged, and it is clear that they cannot anymore be used alone when judging the value of scientific publications (DORA). The value of SM lies in the potential increase in the dissemination of scientific papers (Buckarma et al. 2017), including audiences outside the scientific community and in their acting as a platform for discussion and education (Fox et al. 2016a). We will certainly continue using them and further contribute to researching their impact.

## Electronic supplementary material

Below is the link to the electronic supplementary material.

Supplementary material 1 (DOCX 70 kb)
