# Supplementary material for: If I tweet will you cite later? Follow-up on the effect of social media exposure on article downloads and citations
Source: Int J Public Health. 2020 Nov 7;65(9):1797–802. doi: 10.1007/s00038-020-01519-8 (PMC7716910; doi:10.1007/s00038-020-01519-8)
Supplement: Supplementary file 1 — Supplementary material 1 (DOCX 70 kb) [file 38_2020_1519_MOESM1_ESM.docx]

**International Journal of Public Health**

**If I tweet will you cite later? Follow-up on the effect of social media exposure on article downloads and citations**

**Supplementary Table**

| **Outcome** | **Social Media exposure group (n=65; 3 OA + 62 NOA)** | | | **Control group  (n=65; 6 OA + 59 NOA)** | | | **p-value*** |
| --- | --- | --- | --- | --- | --- | --- | --- |
|  |  |  |  |  |  |  |  |
|  | Counts | Mean (SD) | Median (Range) | Counts | Mean (SD) | Median (Range) |  |
| **Downloads all papers** | 27,812 | 427.9 (345.5) | 312  (153-1932) | 27,496 | 423 (324.1) | 314  (136-1655) | 0.84 |
| **Downloads open access papers** | 4,465 | 1488.3 (386.7) | 1310 (1223-1932) | 5,927 | 987.8 (515.1) | 1028.25 (320-1655) | 0.20 |
| **Downloads non open access papers** | 23347 | 376.6 (249.7) | 303  (153-1740) | 21,569 | 365.6 (238.1) | 283  (136-1544) | 0.61 |
| **Citations all papers** | 267 | 4.11 (3.88) | 3 (0-21) | 237 | 3.65 (2.93) | 3 (0-12) | 0.70 |
| **Citations open access papers** | 28 | 9.33 (10.41) | 6 (1-21) | 26 | 4.33 (2.16) | 4.5 (1-7) | 0.60 |
| **Citations non open access papers** | 239 | 3.85 (3.29) | 3 (0 – 13) | 211 | 3.58 (3.00) | 3 (0 – 12) | 0.63 |

* Wilcoxon rank sum test for difference between groups

**Supplementary Figure**

Relationship between numbers of downloads and number of citations in the SM exposure group (Spearman's rho = 0.47, p= 0.0001) and the control group (Spearman's rho = 0.19, p=0.13) (For both groups: Spearman's rho = 0.32, p= 0.0002). International Journal of Public Health, for articles published between December 2012 and December 2014; downloads and citations for these articles between December 2012 and December 2016

0

5

10

15

20

number of citations

0

500

1000

1500

2000

number of downloads

intervention

control
